# Electroacupuncture-mediated lncRNA TUG1 regulates the miR-127-3p/NF-κB p65 axis to inhibit IBS-D low-grade intestinal inflammation

**DOI:** 10.3389/fimmu.2026.1764684

**Published:** 2026-07-28

**Authors:** Kuiwu Li, Jiaojiao Wang, Ling Zou, Xiaoge Song, Tingting Tong, Jinyu Chen, Shanshan Zhu, Jingwei Zhu, Yu Wang, Haoran Chu

**Affiliations:** 1 The Second Affiliated Hospital, Anhui University of Chinese Medicine, Hefei, China; 2 Xiaodian District People’s Hospital of Taiyuan, Taiyuan, China; 3 Institute of Acupuncture-Moxibustion and Meridians, Anhui University of Chinese Medicine, Hefei, China; 4 Center for Xin'an Medicine and Modernization of Traditional Chinese Medicine, Institute of Health and Medicine, Hefei Comprehensive National Science Center, Anhui University of Chinese Medicine, Hefei, China; 5 Anhui Clinical Medical Research Center of Acupuncture and Moxibustion, Hefei, China

**Keywords:** irritable bowel syndrome, electroacupuncture, long non-coding RNA Taurine Upregulated Gene 1 (lncRNA TUG1), miRNA-127/NF-κB p65, low-grade inflammation

## Abstract

**Background:**

Electroacupuncture (EA) demonstrates efficacy in alleviating diarrhea-predominant irritable bowel syndrome (IBS-D), yet its mechanisms concerning low-grade intestinal inflammation remain insufficiently elucidated. This study investigated whether EA ameliorates IBS-D symptoms by modulating the long non-coding RNA Taurine Upregulated Gene 1 (lncRNA TUG1)/microRNA-127 -3p (miR-127-3p)/nuclear factor-kappa B p65 (NF-κB p65) axis.

**Methods:**

An IBS-D rat model was established using maternal separation, acetic acid enema, and chronic restraint stress. Rats were randomly allocated into control, model, EA (at ST-25 and ST-37), drug (rifaximin), and PDTC (NF-κB p65 inhibitor) groups. Behavioral assessments (body weight, loose stool rate, abdominal withdrawal reflex) were conducted. Molecular analyses included dual-luciferase reporter assays, RT-qPCR, western blot, ELISA, and immunofluorescence to evaluate the TUG1/miR-127-3p/NF-κB p65 axis, inflammatory factors (TNF-α, NLRP3, IL-6), and tight junction proteins (occludin, claudin-1, ZO-1). Intestinal ultrastructure was examined by electron microscopy.

**Results:**

EA significantly improved general status, reduced diarrhea and visceral hypersensitivity in IBS-D rats, comparable to rifaximin and PDTC. Mechanistically, EA upregulated colonic lncRNA TUG1 expression, which sequesters miR-127-3p and partially derepresses NF-κB negative regulators (IκBα, A20), consistent with attenuated NF-κB pathway activation. This was associated with downregulated downstream pro-inflammatory mediators (NF-κB p65, TNF-α, NLRP3, IL-6) in serum and colon tissues. Furthermore, EA restored intestinal barrier integrity, as evidenced by improved mucosal ultrastructure and increased expression of tight junction proteins.

**Conclusion:**

EA alleviates low-grade intestinal inflammation and visceral hypersensitivity in IBS-D rats. The therapeutic effect is mediated, at least in part through upregulation of lncRNA TUG1, which sponges miR-127-3p to inhibit the NF-κB p65 signaling pathway, thereby reducing inflammatory cytokine release and repairing the intestinal epithelial barrier. These findings suggest the TUG1/miR-127/NF-κB axis as a candidate therapeutic target for EA in IBS-D.

## 1 Introduction

Irritable bowel syndrome (IBS) represents a common type of functional gastrointestinal disorder (FGID). Globally, approximately 20% of the general population is impacted by IBS, which substantially influences both quality of life and healthcare expenses (1). Among the four IBS subtypes, the diarrhea-predominant form, IBS-D, is one of the most common and represents a particularly suitable model for investigating the anti-inflammatory mechanisms of EA, because low-grade intestinal inflammation is most consistently documented in this subtype compared to IBS-C or IBS-M. The cardinal symptoms of IBS-D—visceral hypersensitivity and diarrhea—are directly linked to NF-κB p65-driven inflammatory mediator release, creating a clear mechanistic target (2, 3). Recent advances in IBS-D therapeutics have expanded the available repertoire to include pharmacological agents such as rifaximin, antispasmodics, neuromodulators, and bile acid sequestrants, alongside non-pharmacological strategies including low-FODMAP diets, cognitive behavioral therapy, gut-directed hypnotherapy, and acupuncture. Despite this diversification, long-term clinical outcomes remain unsatisfactory due to adverse drug reactions, high treatment costs, and frequent disease recurrence, underscoring the urgent need for mechanism-based interventions with durable efficacy and minimal side effects (4, 5) Concurrently, mechanistic advances have repositioned low-grade intestinal mucosal inflammation as a central pathophysiological pillar, with the gut–brain axis, immune cell activation, barrier dysfunction, visceral hypersensitivity, and gut microbiota dysbiosis collectively driving symptom chronification. Within this network, NF-κB p65 signaling serves as a canonical molecular nexus that translates immune triggers into downstream cytokine release and epithelial barrier compromise, making it a particularly attractive target for mechanism-based therapy.

Acupuncture involves inserting fine needles into specific acupoints to modulate meridian and qi flow, producing anti-inflammatory and therapeutic effects. Acupuncture has shown efficacy across numerous systemic diseases and is emerging as a promising non-pharmacological approach for FGIDs (6). In multiple randomized controlled trials, acupuncture has been shown to be more effective than certain medications for IBS treatment. Its therapeutic effects may persist for up to 12 weeks, substantially alleviating symptoms like abdominal pain and diarrhea (7–9). These studies suggest that acupuncture has beneficial, long-term effects in the treatment of IBS-D. Nevertheless, the molecular biological mechanism in IBS-D is not fully understood, and the anti-inflammatory mechanism of acupuncture in IBS-D also remains to be fully explained.

In the context of IBS-D pathophysiology, low-grade intestinal mucosal inflammation caused by mucosal barrier injury is central to the condition, which is associated with the onset and progression of abdominal pain, diarrhea, visceral hypersensitivity, and other clinical manifestations in patients (10–12). Disruption of the intestinal epithelial barrier in IBS-D is associated with immune cell activation and release of inflammatory mediators (13), contributing to low-grade inflammation that exacerbates barrier dysfunction and visceral hypersensitivity. The nuclear factor (NF)-κB signaling cascade serves as a canonical pathway involved in inflammatory responses. It significantly contributes to cytokine regulation during inflammation by triggering the expression of downstream inflammatory mediators. This cascade upregulates pro-inflammatory cytokines including interleukin (IL)-6, IL-10, and tumor necrosis factor (TNF)-α, and increases expression of the NOD-like receptor pyrin domain-containing protein 3 (NLRP3) inflammasome (14, 15). Electroacupuncture (EA), a widely utilized form of acupuncture, combines conventional acupuncture techniques with low-intensity electrical stimulation. This approach is utilized to alleviate inflammation and mitigate pain (16). We selected ST25 (Tianshu, the front-mu point of the large intestine) and ST37 (Shangjuxu, the lower-he-sea point of the same meridian). This “He-Mu combination, ” is a canonical principle for treating disorders of the fu-organs (hollow viscera). This specific pairing is intended to harmonizes Yin-Yang, unblocks fu-qi and arrests diarrhea according to traditional theory, and contemporary studies show that EA at ST25 and ST37 suppresses intestinal inflammation and reduces visceral hypersensitivity in IBS-D (17, 18). EA was delivered at ST25 and ST37 using alternating dense-sparse frequencies (15 Hz/2 Hz), parameters selected to maximize neuromodulatory and anti-inflammatory effects. This study investigates EA as an integrated clinical protocol rather than dissecting the individual contributions of acupoint selection, needle insertion, or electrical stimulation. Our objective is to identify the downstream molecular pathways engaged by this standardized intervention. Previous investigations have established that EA inhibits NF-κB p65 signaling in IBS-D models, reducing pro-inflammatory cytokines and restoring barrier function (19). However, the upstream molecular mechanisms initiating this NF-κB p65 suppression remain undefined. Specifically, whether EA modulates NF-κB p65 through direct interference with pathway components or via upstream regulatory networks involving non-coding RNAs has not been investigated. The present study addresses this critical gap by identifying the lncRNA TUG1/miR-127-3p axis as a previously unrecognized upstream regulator that links EA intervention to NF-κB p65 inhibition in IBS-D.

Long non-coding RNAs (LncRNAs) are potential key regulators of the inflammatory response (20). According to research reports, the long non-coding RNA taurine up-regulated gene 1 (TUG1) is associated with IBS-D and can reduce inflammation in gastrointestinal diseases (21, 22). There was a negative correlation between microRNA-127-3p (miR-127-3p) and the lncRNA TUG1. miR-127-3p has a proinflammatory effect (23) and can activate the NF-κB p65 signaling pathway (24), triggering inflammatory responses. Therefore, the miR-127-3p/NF-κB p65 axis is closely associated with inflammation. As the potential key regulators of the inflammatory response, lncRNA TUG1 exerts a key effect on reducing inflammation in gastrointestinal diseases. We selected the TUG1/miR-127-3p axis based on converging evidence. TUG1 is significantly downregulated in IBS-D patient serum and correlates negatively with disease severity. miR-127-3p promotes macrophage inflammation and activates NF-κB p65 signaling. Bioinformatic predictions identified direct binding between TUG1 and miR-127-3p, suggesting TUG1 functions as a competing endogenous RNA to sponge miR-127-3p and suppress NF-κB p65 activity. While this axis has been studied in other diseases, its relevance to IBS-D intestinal mucosal inflammation and modulation by EA was previously unexplored.

Although prior studies have demonstrated that EA suppresses NF-κB p65 activity and attenuates downstream pro-inflammatory mediators in IBS-D models, this body of work has focused predominantly on descriptive efficacy or downstream pathway inhibition without identifying the upstream molecular triggers that initiate this suppression. Specifically, it remains entirely unknown whether EA modulates NF-κB p65 through direct interference with pathway components or via upstream regulatory networks involving non-coding RNAs. The present study addresses this critical gap by examining the lncRNA TUG1/miR-127-3p axis as a previously unexplored upstream mechanism that links peripheral EA stimulation to NF-κB p65 inhibition and subsequent mucosal anti-inflammatory effects in IBS-D. Aiming to establish a more comprehensive scientific basis for using EA in treating low-grade intestinal inflammation in IBS-D.

## 2 Materials and methods

### 2.1 Animals

The experimental protocols were conducted in strict compliance with national regulations governing the humane treatment and utilization of laboratory animals. Prior to implementation, formal approval was obtained from the Animal Ethics Committee at Anhui University of Chinese Medicine (ref. AHUCM-RATS - 2024190). Seven adult pregnant Sprague-Dawley (SD) rats (body weight: 360 ± 20 g) were acquired from the Animal Research Facility at Anhui Medical University [animal license no. SCXK(Anhui)2017-001]. The instrument and reagent specifications are provided in **Supplementary Table 1**.

All rats were acclimatized to the housing conditions for 7 days prior to any experimental procedures. These experimental animals were subsequently maintained in the controlled-environment vivarium of the Laboratory Center at Anhui University of Chinese Medicine throughout the study period. The photoperiod was maintained at 12 hours of light and 12 hours of darkness. The ambient temperature was kept at 22 ± 2 °C, with a relative humidity range of 50-70%. Ad libitum access to food and water was provided for the animals. The study design adhered to the principles of animal welfare, with rigorous measures implemented to reduce both the number of animals utilized and any potential distress they might experience.

### 2.2 Cell lines

HEK-293T cell line was used for this study. Cells were cultured in Dulbecco’s Modified Eagle Medium (DMEM) supplemented with 10% fetal bovine serum (FBS) and maintained in a humidified incubator at 37 °C with 5% CO_2_. All experiments were performed with cells at a low passage number and during the exponential growth phase. The cell lines present in this study were obtained from ICell Bioscience Inc.

### 2.3 Establishment of the IBS-D animal model

The IBS-D rat model was developed through combining maternal and neonatal maternal separation, acetic acid (AA) enema, and wrap restraint stress (25). Following delivery, a total of 28 female pups (mean birth weight 5.81 ± 0.43 g) and 33 male pups (mean birth weight 6.05 ± 0.41 g) were obtained. One male pup died of severe weakness; the remaining 60 pups were used for model establishment. From postnatal day 2 onward, litters were separated from the dam for 3 h daily, beginning at 09:00, and then returned to their home cage. Mother and child separation was not carried out on PND 15, and all suckling rats were fed normally until PND 24 when they were weaned and taken out. Starting from PND 30, male and female rats were segregated into distinct cages. On the PND 35, the model rats underwent a 24-hour fast, but water was allowed. On PND 36 to 38, their rectum was stimulated with AA. After deep anesthesia with isoflurane inhalation (induction 4%, maintenance 2%), 1 mL of 4% AA was injected into the colon 4–5 cm away from the anus by connecting a syringe with a central intravenous catheter (1 mm). The rats were then placed on their heads, stimulated for 60 s, and washed with 1 mL of 0.01 M phosphate buffered solution (PBS). On PND 39 to 45, the rats’ forelimbs and upper bodies were bound with paper adhesive tape, with only the head being able to swing, 2 hours a day, for a total of 1 week. After the experimental procedures, the successful establishment of the IBS-D rat model was validated in accordance with three sets of specific criteria (26): the mean body weight gain as an indicator of overall physical condition, visceral sensitivity was assessed via abdominal withdrawal reflex (AWR) scores, and the rate of loose stools per 6 hours to evaluate the severity of diarrhea.

### 2.4 Animal groups and treatments

To eliminate litter effects and ensure inter-group comparability, littermates were sex-stratified immediately after birth and randomly allocated to experimental groups so that each group received an equivalent number of males and females from multiple litters. Both male and female rats were included in the final analysis, and data were pooled across sexes. Sex was balanced across groups by stratified randomization. A total of 60 suckling rats were randomly allocated into five groups, with 12 rats in each group. The groups were as follows: the control group, the model group, the EA group, the drug group (treated with rifaximin), and the PDTC group (treated with pyrrolidine dithiocarbamate, which is a NF-κB p65 antagonist). No rats failed modeling during the experiment.

The rats in the drug group were given rifaximin (0.2 g/tablet, Alpha Sigma Co., Ltd., national drug approval WORD H20181212), which was converted according to the “Table of Equivalent Dose conversion coefficients of different animals, ” that is, daily dose of rats (weight 200 g) = daily dose of human (weight 50 kg) × conversion coefficient R, r = 0.018. Rifaximin was ground into a fine powder by gavage at 150 mg/kg/d (27). According to the weight of each rat, the corresponding powder was weighed using an electronic scale, and a suspension (2 mL) was prepared with distilled water once a day, treatments began on PND 47, two days after the final restraint stress session, and continued for 7 days.

The rats in the PDTC group were given PDTC (Shanghai Yuanye Biotechnology Co., Ltd., S30342) at 50 mg/kg/d (28). PDTC (powder) was dissolved in 0.9% sodium chloride solution and prepared into a solution with a concentration of 10 mg/mL. The rats were injected intraperitoneally with the solution once a day, treatment timing and duration were as described above. A control injection of saline was used in the other groups.

The Control and Model groups received intraperitoneal injection saline (equal volume to the PDTC group), intragastric distilled water (equal volume to the Drug group), and grasping/fixation (synchronized with the EA group). The PDTC group received intragastric distilled water and grasping/fixation. The Drug group received an intraperitoneal injection saline and grasping/fixation. The EA group received intraperitoneal injection saline and intragastric distilled water.

Under light isoflurane anesthesia (29), the rats in the EA group were fixed on a fixation plate. First, the skin overlying the sites of needling was disinfected with 75% ethanol. Stainless steel acupuncture needles (Tianxie, diameter 0.25 mm x length 25 mm; Suzhou Tianxie Instrument Co., Ltd., China) were inserted to a depth of 3–5 mm at ST25 (*Tianshu*) and ST37 (*Shangjuxu*) bilaterally. ST25 is on a horizontal line 5 cun above the pubic symphysis and 2 cun away from the midline. ST37 is situated 5 mm from the anterior tubercle of the tibia and 20 mm distant from the knee joint (30, 31). EA was administered using alternating dense-sparse frequency patterns (15 Hz for 1.5 s and 2 Hz for 1.5 s, alternately, pulse width 0.3 ms, intensity 2 mA) (32). An individual EA session was administered daily for 15 min, treatment commenced on PND 47 and continued for 7 days as described for the other intervention groups (26). EA treatment commenced on PND 47, two days after the final restraint stress session. This timing was selected for three reasons: (i) acute stress responses had resolved while the chronic IBS-D phenotype (confirmed at PND 45) remained stable; (ii) EA was evaluated as a disease-modifying intervention in established pathology, approximating clinical treatment scenarios; and (iii) the post-weaning juvenile stage aligns with the human adolescent/young adult demographic relevant to early-life stress-related IBS-D.

**Figure 1:**
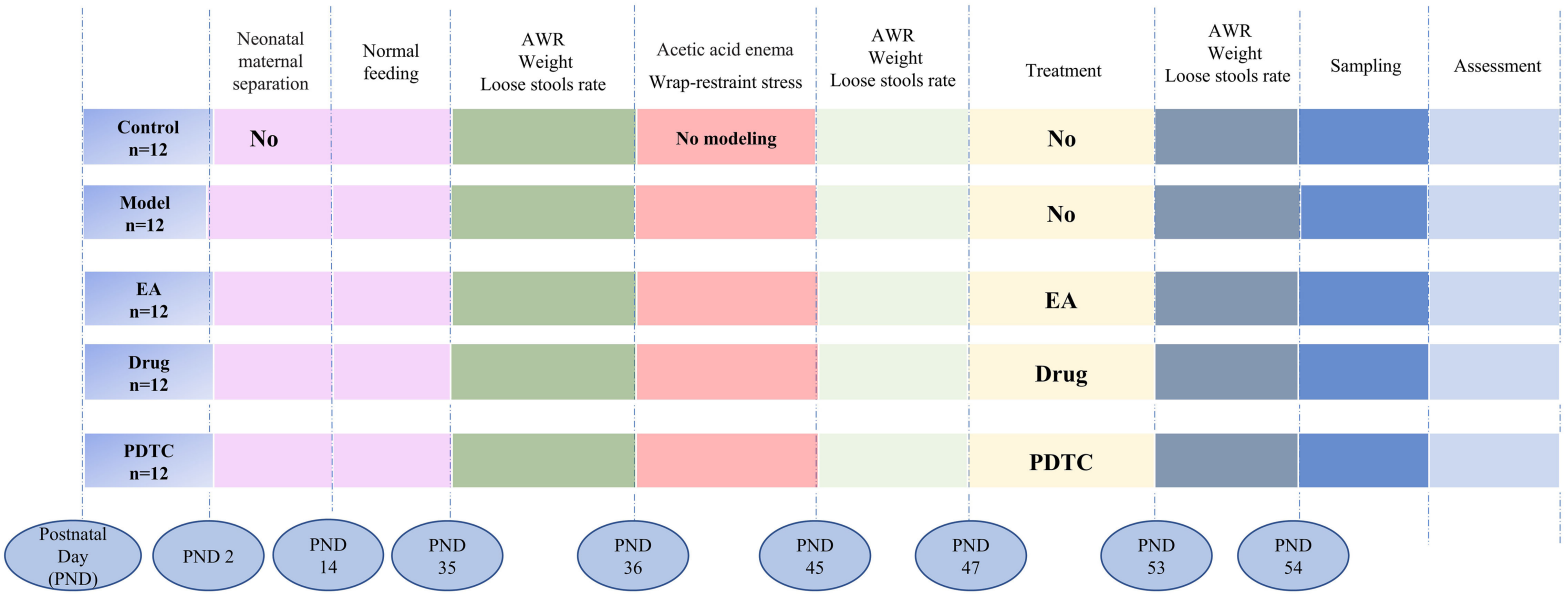
Flow chart of the experiment. intervention timeline for the control (Control), model (model), electroacupuncture (EA), drug (Drug), and PDTC (PDTC) groups.

Random allocation sequences were generated by an investigator not involved in outcome assessment, and coded ear tags were affixed. During modelling and treatment, operators performing EA, gavage, or PDTC injection were unaware of group identity. Behavioral scoring, histology, ELISA, qPCR, and Western blot evaluation were performed by a second investigator blinded to group codes, while statistical analyses were conducted by a third independent researcher. Codes were broken only after the final dataset was locked. Intervention practitioners were necessarily unblinded due to procedural requirements, but all outcome assessors and analysts remained masked to group assignments. The experimental design is shown in **Figure 1**.

### 2.5 General status assessment

Before modeling (PND 35), after modeling (PND 45), and after EA intervention (PND 53), biological characteristics (behavior, hair status, and body weight) were observed, as well as the loose stool rate of the rats in each group.

#### 2.5.1 Body weight

The rats in each group were weighed using an electronic balance and the values were recorded. Three measurements of each rat’s weight were obtained, and the average was calculated.

#### 2.5.2 Loose stool rate determination

The rats were placed alone in a gridiron cage(6 h per day), and a plastic tray with filter paper was placed underneath. The filter paper was replaced once every 1 h. Within a 6-hour time frame, two separate researchers independently documented both the total quantity of feces and the specific count of loose feces, and the rate of loose feces was calculated three times. Loose defecation rate = number of loose feces ÷ total number of feces × 100% (33).

### 2.6 Colonic visceral sensitivity test

To evaluate the colonic visceral sensitivity of rats, AWR scores were measured by noninvasive colorectal balloon dilation. When the AWR score reaches 3, the minimum amount of water pushed into the colon of the rats was recorded to assess the sensitivity of the colon organs. Isoflurane inhalation (induction 4%, maintenance 2%) was used for anesthesia. The injection volume and AWR scores were assessed and quantified via an published method (34).

### 2.7 Sample collection and processing

All tissue samples were collected on postnatal day 54 (PND 54), 24 h after the final treatment session. Six rats were selected by the random number table method. Colon tissues were obtained for real-time quantitative polymerase chain reaction (RT–qPCR) and Western blotting (WB), while blood samples were collected for enzyme-linked immunosorbent assay (ELISA). In the remaining 6 rats, after internal fixation, colon tissue samples were collected and processed for hematoxylin and eosin (HE) staining as well as immunofluorescence analysis.

Rats were deeply anaesthetized with 2% pentobarbital sodium (2 mL/kg, i.p.), and 4–5 mL of whole blood was taken from the abdominal aorta for ELISA, and immediately euthanized by cervical dislocation while still under deep anesthesia. Afterward, about 2–3 cm of colon tissue was swiftly removed from each rat, kept in the refrigerator at –80 °C for later RT–qPCR and WB procedure. The remaining six rats per group underwent transcardial perfusion with 200 mL of physiological saline (0.9% NaCl), followed by 300 mL of 4% paraformaldehyde solution prepared in phosphate-buffered saline (PBS, 0.1 M concentration, pH 7.4). Following perfusion completion, excised colonic specimens were split into two parts, and one part was immediately immersed in fresh 4% paraformaldehyde-PBS fixative (pH-adjusted to 7.4) for optimal tissue preservation. and then paraffin-embedded for HE staining and immunofluorescence detection. After cutting the other vertically, trim it into two rectangular prisms measuring 1 mm × 1 mm × 2 mm, and place them in the 2.5% glutaraldehyde solution (Cat# 2200218, Ted Pella, Inc.), used for electron microscopy observation.

### 2.8 Hematoxylin-eosin staining

Colon tissues fixed in paraffin were sectioned at a thickness of 4 μm, dewaxed, stained with HE, and mounted with neutral mounting medium. An optical microscope (×200, Olympus, Japan) was employed to evaluate the histopathological alterations, such as edema, erosion, and inflammatory cell infiltration, and the images were collected. Histological damage score (HDS): Hematoxylin-eosin (HE)-stained colonic sections were assessed with a standardized histological damage scoring system. Two blinded pathologists independently selected five non-overlapping 400× high-power fields per sample, and average scores were used for intergroup comparison. The total HDS (0–8 points) combined epithelial damage and inflammatory infiltration (each scored 0–4 points) (35). Epithelial score: 0 = normal structure; 1–2 = partial goblet cell loss; 3 = crypt distortion/focal loss; 4 = extensive crypt loss or ulceration. Infiltration score: 0 = no inflammation; 1 = mild pericrypt infiltration; 2 = moderate infiltration into the muscularis mucosae; 3 = marked mucosal inflammation with edema; 4 = severe transmural infiltration to the submucosa. Inflammatory cell count: Five random 400× fields per HE section were photographed with a calibrated Olympus light microscope. Total inflammatory cells, neutrophils, and lymphocytes were counted manually in each field. Results were presented as cells per high-power field (cells/HPF), with mean values from five fields used for statistical analysis. Strict controls were applied to ensure reliability: random field selection via grid overlay to avoid bias; independent scoring by two pathologists with consistency evaluation; group information concealed during analysis and decoded only after data collection; unified calibration of microscope magnification and image capture for all samples.

### 2.9 Dual luciferase reporter assay

The BiBiServ RNAhybrid tool (https://bibiserv.cebitec.uni-bielefeld.de/rnahybrid;jsessionid=569fee10c71f91ef6bd99aced0d6) was employed to identify the binding sites between (i) rno-lncRNA TUG1 and rno-miR-127-3p, (ii) rno-miR-137-3p and NF-κB p65, as well as (iii) hsa-rno-miR-127-3p and its putative targets hsa-IκBα, hsa-A20, and hsa-CYLD. The interaction of target genes was verified employing a dual-luciferase reporter assay. Briefly, utilizing pmirGLO vectors (E1330, Promega, USA), we synthesized recombinant plasmids for the luciferase reporter. In the subsequent experiments, the 293T cell line was transfected using luciferase reporter recombinant plasmids along with rno-miR-127-3p mimic or NC. Relative luciferase activities were measured by means of dual-luciferase detection kits (E1910, Promega, USA).

### 2.10 ELISA

The whole blood samples were subjected to centrifugation at 700 g for 15 minutes at 4°C. After this step, the upper serum was collected and assessed with the aid of ELISA kits (NF-κB P65, JYM0205Ra; TNF-α, JYM0635Ra; IL-6, JYM0646Ra; NLRP3, JYM1110Ra. Wuhan Genmei Biotechnology Co., Ltd., China). The expression levels of the NF-κB P65, TNF-α, NLRP3, and IL-6 inflammatory factors in serum were measured using a double-antibody sandwich method, following the manufacturer’s protocols. Lastly, the absorbance of each well was monitored at a wavelength of 450 nm.

### 2.11 Protein extraction and western blotting

Colon tissue was ground thoroughly in liquid nitrogen. Then it was lysed with radio immunoprecipitation assay (RIPA) lysis buffer (P0013B, Beyotime, Shanghai, China), homogenized, and centrifuged for 15 min at 4°C and 10000 g. The supernatant, which encompasses the total protein, was recovered, and via utilization of a bicinchoninic acid (BCA) assay kit, the concentration of the protein was ascertained. After cooling, the volume was calculated according to the concentration. A quantity of 40 μg of total protein extracted from each rat underwent analysis via dodecyl sulfate, sodium salt (SDS)-polyacrylamide gel electrophoresis (SDS-PAGE). Subsequently, the samples were transferred onto polyvinylidene fluoride membranes and subjected to blocking with 5% skimmed milk at room temperature for a duration of 2 hours. Following this, the samples were incubated with the primary antibody and then the secondary antibody overnight at room temperature for 1.5 h, followed by development with an enhanced chemiluminescence (ECL) kit (34094, Thermo Scientific, Waltham, MA, USA). The film strip was analyzed with the aid of ImageJ software (National Institutes of Health, Bethesda, MD, USA). The relative expression level of the target protein was assessed by determining the ratio of its gray value to that of β-actin or GAPDH.

### 2.12 RNA isolation and quantitative reverse transcription PCR

The mRNA expression levels of lncRNA TUG1, rno-miR-127-3p, NF-κB p65, TNF-α, IL-6, NLRP3, IκBα, A20, and CYLD in the colon were measured by RT-qPCR. Total RNA was extracted from colon tissues, assessed for purity, and reverse transcribed into cDNA for PCR amplification. The primer sequences are detailed in **Table 1**.

**Table 1:** RT-qPCR primer sequences for target genes.

Gene	Forward primer(5’→3’)	Reverse primer(5’→3’)
β-actin	CCCATCTATGAGGGTTACGC	TTTAATGTCACGCACGATTTC
lncRNA TUG1	TTATTCCCTCCTGCCATAC	TTGGGTGATACTGTTGATTTAG
rno-miR-127-3p	GCTCGGATCCGTCTGAGC	AGTGCAGGGTCCGAGGTATT
rno-miR-127-3p-RT	GTCGTATCCAGTGCAGGGTCCGAGGTATTCGCACTGGATACGACAGCCAA	
NF-κB p65	TCTACAGGCAGAAGGCGGAGGA	TGGCGTCTGACACCACAGGTTC
TNF-α	GGGCCACCACGCTCTTCTGT	GGCTACGGGCTTGTCACTCG
IL-6	GCCTTCTTGGGACTGATGTT	ACTGGTCTGTTGTGGGTGGT
NLRP3	ATGCCGTATCTGGTTGTGTT	GACTTAGGAAGAGCTGAGCA
IκBα	GAAAATCTGGGGAAACTCAGC	TGTTAAATAGCCACACCCCG
A20	ACGACCTTTGCTTACTATGT	AATGGCAGAAACCTCCTC
CYLD	TTTTGCCAGCTAATGTTC	TACTGTCCTATGCTCCCT

### 2.13 Immunofluorescence

The colon tissue sections were successively subjected to xylene for 5 min three times. Then, the samples were passed through ethanol in decreasing concentrations (100% 95%, and 80%, respectively) for 3 min per pass. Then, 2000 ml EDTA repair solution (pH = 8.0) was prepared in a high-pressure pot, heated to boiling in an induction furnace and placed into the pot. The pot was then covered and, 2 min after air injection, the heat was turned off and the high-pressure pot cover was slowly rinsed under running water until it cooled. Each section was circled with an immunohistochemical brush and placed in an incubator box. Goat serum (ZLI-9022, Zsbio, China) was sealed for 30 min and then removed. The primary antibodies were applied to the sections, which were then incubated at 37 °C for 1 hour. Subsequently, the sections underwent three washes in PBS, and dried by shaking. The samples were then incubated with fluorescent secondary antibody, at 37 °C for 30 min, washed with PBS three times, and again dried by shaking. In addition, the sections underwent restaining with 4’, 6-diamidino-2-phenylindole (DAPI) (P0126, Beyotime, China) for a duration of 5 minutes. Following this, they were subsequently rinsed three times with PBS. After washing, the sections were mounted using anti-fluorescence quenching tablets (P0126, Beyotime). Finally, imaging was performed on these prepared sections with a fluorescence microscope (BA410E, Motic, China). Fluorescence microscopy shows the nucleus as blue, and the positive expression position as green light. CaseViewer 2.4 software was used to read and take screenshots. The mean optical density for positive expression was analyzed using the Jetta JD801 morphological image analysis system (JEDA, Nanjing, China).

### 2.14 Transmission electron microscopy

Remove the colon tissue from the 2.5% glutaraldehyde solution, and sequentially wash it with PBS, fix it with osmium acid, wash it with PBS followed by gradient ethanol dehydration, acetone infiltration, embedding solution infiltration, and embedding. Prepare ultra-thin sections (thickness 70 nm) and stain them with uranyl peroxide and lead citrate double staining for 15 minutes each. Dry at room temperature overnight and observe the microvilli and tight junctions of colon mucosal epithelial cells under transmission electron microscopy (HT7700, Hitachi, Japan).

### 2.15 Scanning electron microscopy

Remove the colon tissue from the 2.5% glutaraldehyde solution, and sequentially wash it with PBS, fix it with osmium acid, perform gradient ethanol dehydration after PBS washing, replace it with hexamethyldisilazane, and then vacuum dry it. Use an ion sputtering instrument to coat and spray gold, and observe the ultrastructure of the colonic mucosa under a scanning electron microscope (JSM-6701F, Hitachi, Japan).

### 2.16 Statistical analysis

IBM SPSS 26.0 was used for statistical analysis, Meanwhile, GraphPad Prism 8 was employed to create the graphs. The mean ± standard deviation (SD) is used to represent measurement data. Repeated measures multivariate analysis of variance (MANOVA) was used to analyze body weight, sparse bowel rate, and minimum volume threshold of the noninvasive colorectal dilatation reflex. The correction was conducted using the Greenhouse–Geisser method. Simple effects analysis was performed if an interaction was detected. For the remaining measurement data, one-way ANOVA was adopted if the measurement data had uniform variance; otherwise, Welch´s ANOVA was adopted. For correlations, if two parameters had a normal distribution, bivariate Pearson correlation analysis was adopted; if not, Spearman correlation analysis was used. A statistically significant difference was indicated when *P* < 0.05.

## 3 Results

### 3.1 EA ameliorates general status, visceral hypersensitivity, and colonic mucosal histopathology in IBS-D rats

#### 3.1.1 EA improves the general status of IBS-D rats

Rats in each group had sensitive movement, smooth and clean hair, and normal stool on postnatal day 35 (PND 35, before modeling). On PND 45 (the end of modeling), the state of the rats in the control group was unchanged. After modeling, the rats showed slightly sluggish movement, with withered, yellow, scattered, and dull hair. They preferred to gather in the corner of the cage and had obvious, thin, soft, and shapeless stools. On PND 53 (after treatment), gross visual inspection suggested that rats in the EA, drug and PDTC groups appeared more active, had sleeker coats and formed stools compared with the model group; these subjective observations were subsequently corroborated by the quantitative assays described below.

##### 3.1.1.1 Body weight of rats

**Figure 2:**
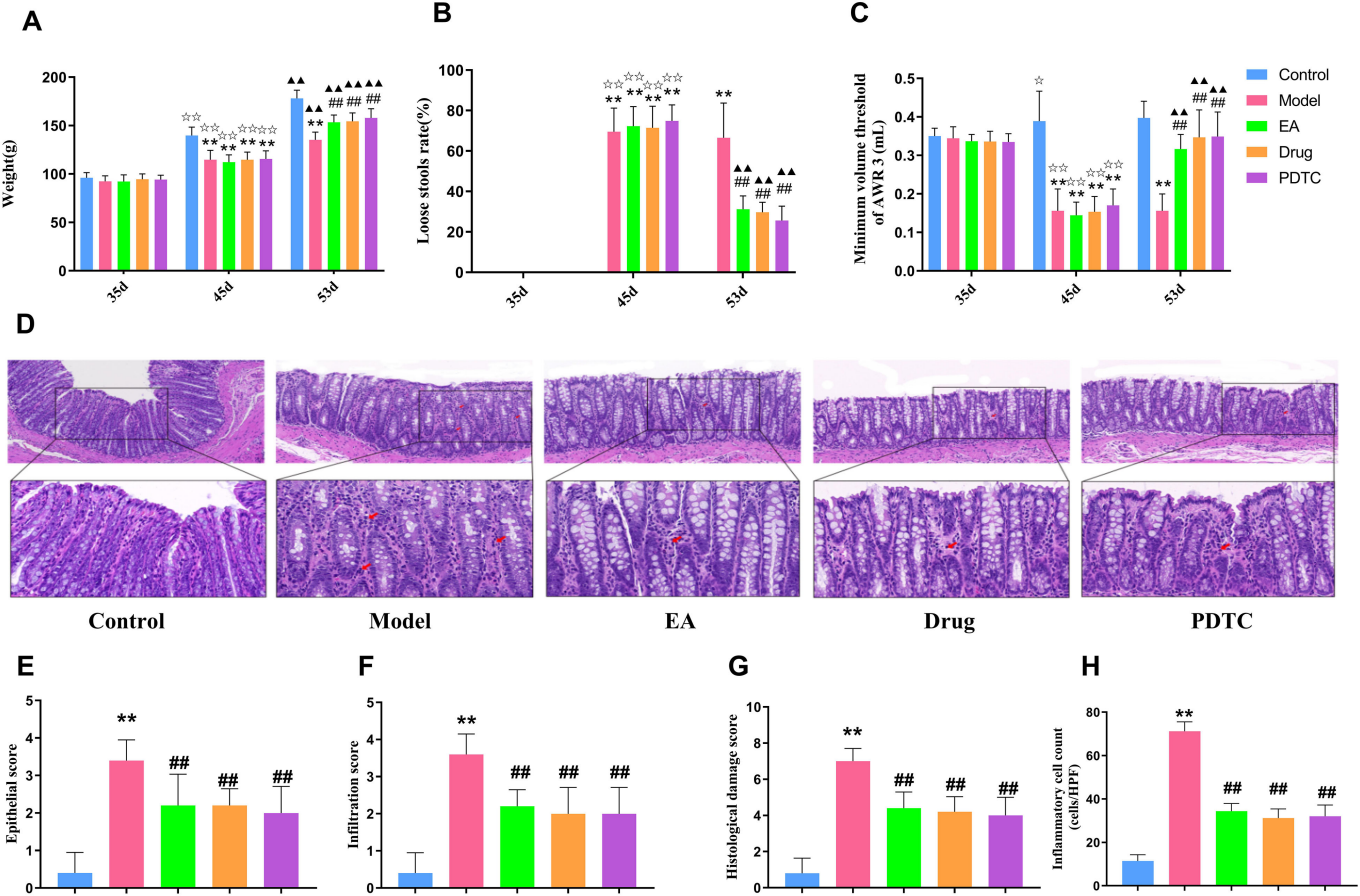
Electroacupuncture reduces diarrhea and visceral hypersensitivity in IBS-D rats, and alleviates the pathological state of intestinal mucosal inflammation. **(A)**. Body weight on PND 35 (before modeling), PND 45 (after modeling), and PND 53 (after intervention). n = 12. **(B)**. Loose stool rate of rats in each group on PND 35 (before modeling), PND 45 (after modeling), and PND 53 (after intervention), n = 12. **(C)**. Minimum volume threshold of AWR up to three points on PND 35 (before modeling), PND 45 (after modeling), and PND 53 (after intervention). n = 12. **(D)**. Micrograph of hematoxylin–eosin staining of rat colon tissue. Scale bar, 20 µm; lower side is the magnified view, scale bar, 50 µm. n = 6. **(E)** Epithelial score; **(F)** Infiltration score **(G)** Histological damage score; **(H)** Inflammatory cell counts (total cells/HPF). n = 6. **p < 0.01 versus the control group, ^##^p < 0.01 versus the model group, ^☆^p < 0.05, ^☆☆^p < 0.01 versus PND 35, ^▲▲^p < 0.01 versus PND 53.

Mean body weights (mean ± SD, g) on the first treatment day were: Control group:148.65 ± 7.36; Model group: 119.81 ± 8.48; EA group: 121.31 ± 7.14; Drug group:123.17 ± 7.42; PDTC group: 123.38 ± 8.06. All drug and EA doses were calculated from these individual weights. At each time point, repeated ANOVA showed that the body weight of rats in each group was significantly different (*F* = 3124.901, *P* < 0.001). In addition, an interaction was observed between body weight and time (*F* = 28.564, *P* < 0.001), with statistical significance at different time points (*F* = 2829.665, *P* < 0.001). After PND 35, simple effects analysis revealed no significant differences in body weight between groups (*P* > 0.05). However, on PND 45 (after modeling), the body weight decreased significantly (*P* < 0.01) in the model, EA, and drug groups compared to the control group. Body weight increased significantly (*P* < 0.01) in the EA, drug, and PDTC groups on PND 53 (after intervention) compared to the model group (**Figure 2A**).

##### 3.1.1.2 Loose stool rate of rats

Repeated ANOVA showed significant differences in the rate of loose stools at each time point (*F* = 1510.078, *P* < 0.001), and an interaction existed between the rate of loose stools and time (*F* = 63.936, *P* < 0.001). The comparison between groups was also statistically significant (*F* = 928.885, *P* < 0.001). The simple effects analysis showed no significant difference in the loose stool rate among all groups after PND 35 (*P* > 0.05). On PND 45, however, the loose stool rate increased significantly (*P* < 0.01) in the model, EA, drug, and PDTC groups compared to the control group. On PND 53, the loose stool rate decreased significantly (*P* < 0.01) in the EA, drug, and PDTC groups compared to the model group (**Figure 2B**).

#### 3.1.2 EA reduces visceral sensitivity in the colon of IBS-D rats

Repeated ANOVA showed that the minimum water injection volume at AWR score 3 in each group was significantly different at each time point (*F* = 140.475, *P* < 0.001), and an interaction existed between the minimum water injection volume at AWR score 3 and time (*F* = 25.286, *P* < 0.001). The comparison between groups was also statistically significant (*F* = 137.583, *P* < 0.001). After simple effects analysis, no significant difference was observed in the minimum water injection volume at AWR score 3 among all groups on PND 35 (*P* > 0.05). However, when compared to the control group on PND 45, the minimum water injection volume at AWR score 3 decreased significantly (*P* < 0.01) in the model, EA, drug, and PDTC groups. In addition, on PND 53, the minimum volume threshold significantly increased (*P* < 0.01) in the EA, drug, and PDTC groups compared to the model group (**Figure 2C**).

#### 3.1.3 EA improves the colonic mucosa pathological characteristics in rats with IBS-D

The histological observation of the colon showed that the epithelial structure of the colonic mucosa of rats in each group was intact, the glands of the colonic mucosa were orderly arranged, and no edema, erosion, or ulcer was observed in the mucosal and serosal layers, indicating no obvious pathological changes in the colonic mucosa of rats in each group. Compared with the control group, the rats in the model group showed increased inflammatory infiltration in the colonic interstitial mucosa (shown with an arrow in **Figure 2D**). After the treatment, the inflammatory infiltration in the colonic interstitial mucosa decreased in the EA and drug groups (**Figure 2D**). Quantitative histological assessment revealed significantly elevated Histological Damage Score in the model group compared to control (*P* < 0.01), characterized by increased inflammatory cell infiltration (*P* < 0.01). EA treatment significantly reduced both HDS (*P* < 0.01 versus model) and inflammatory cell counts (*P* < 0.01 versus model), comparable to rifaximin and PDTC interventions (**Figures 2E–H**).

### 3.2 EA modulates the colonic lncRNA TUG1/miR-127-3p axis

#### 3.2.1 Interaction exists between lncRNA TUG1 and miR-127-3p and between miR-127-3p and NF-κB p65

**Figure 3:**
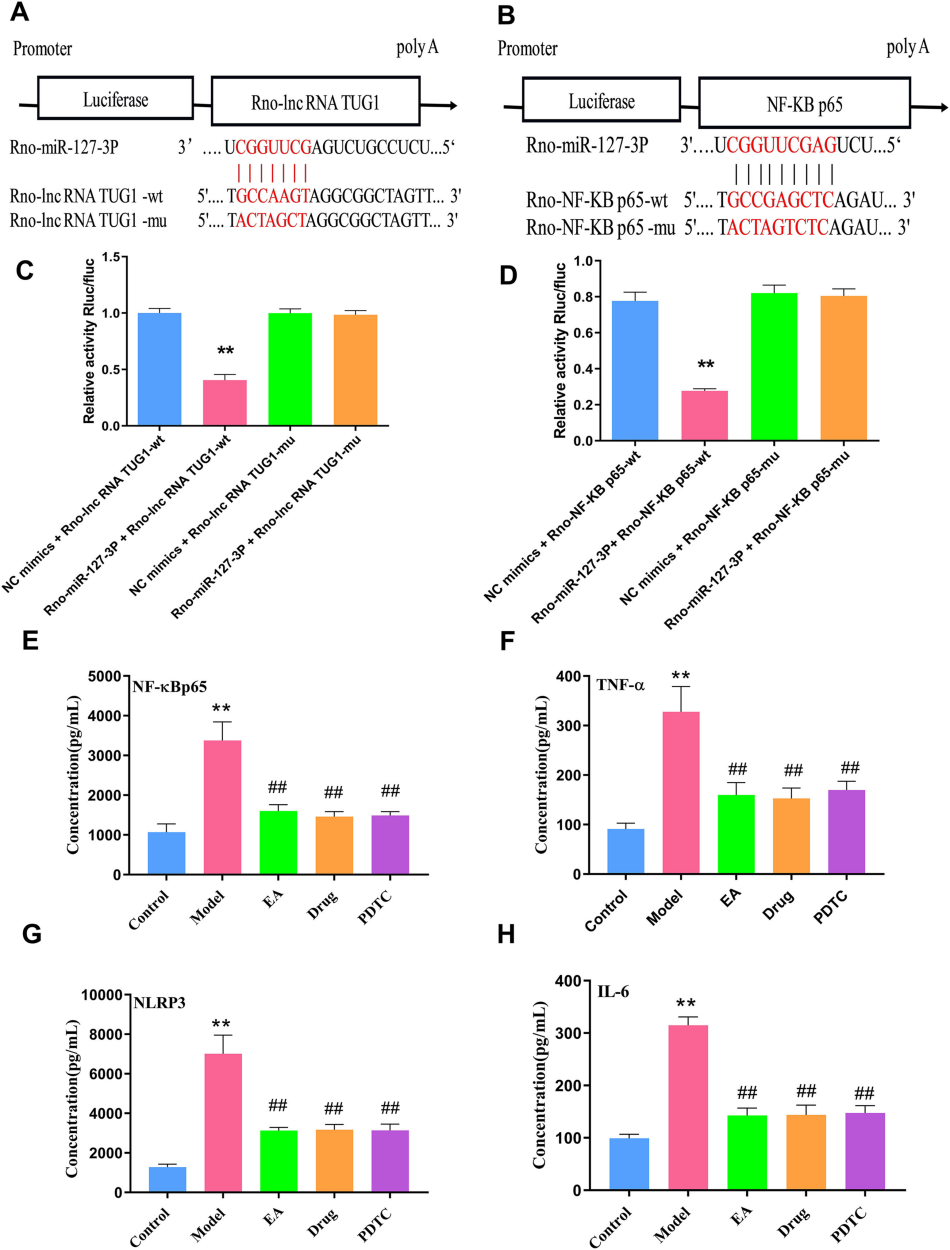
Interaction exists between lncRNA TUG1 and miR-127 and between miR-127 and NF-κ **(B)** Electroacupuncture can reduce the expression of serum NF-κ B P65 and related inflammatory factors in IBS-D rats (n = 6). **(A–D)** Analysis was performed by the Dual Luciferase Reporter Assay. An interaction exists between lncRNA TUG1 and miR-127 **(A, C)**, and between miR-127 and NF-κB **(B, D)**. ** p < 0.01 compared with the NC group. **(E-H)** The expression of NF-κB P65 **(E)**, TNF-α **(F)**, NLRP3 **(G)**, and IL-6 **(H)** in the serum of rats, detected using ELISA. ^**^p < 0.01 versus the control group, ^##^p < 0.01 versus the model group.

**Figure 4:**
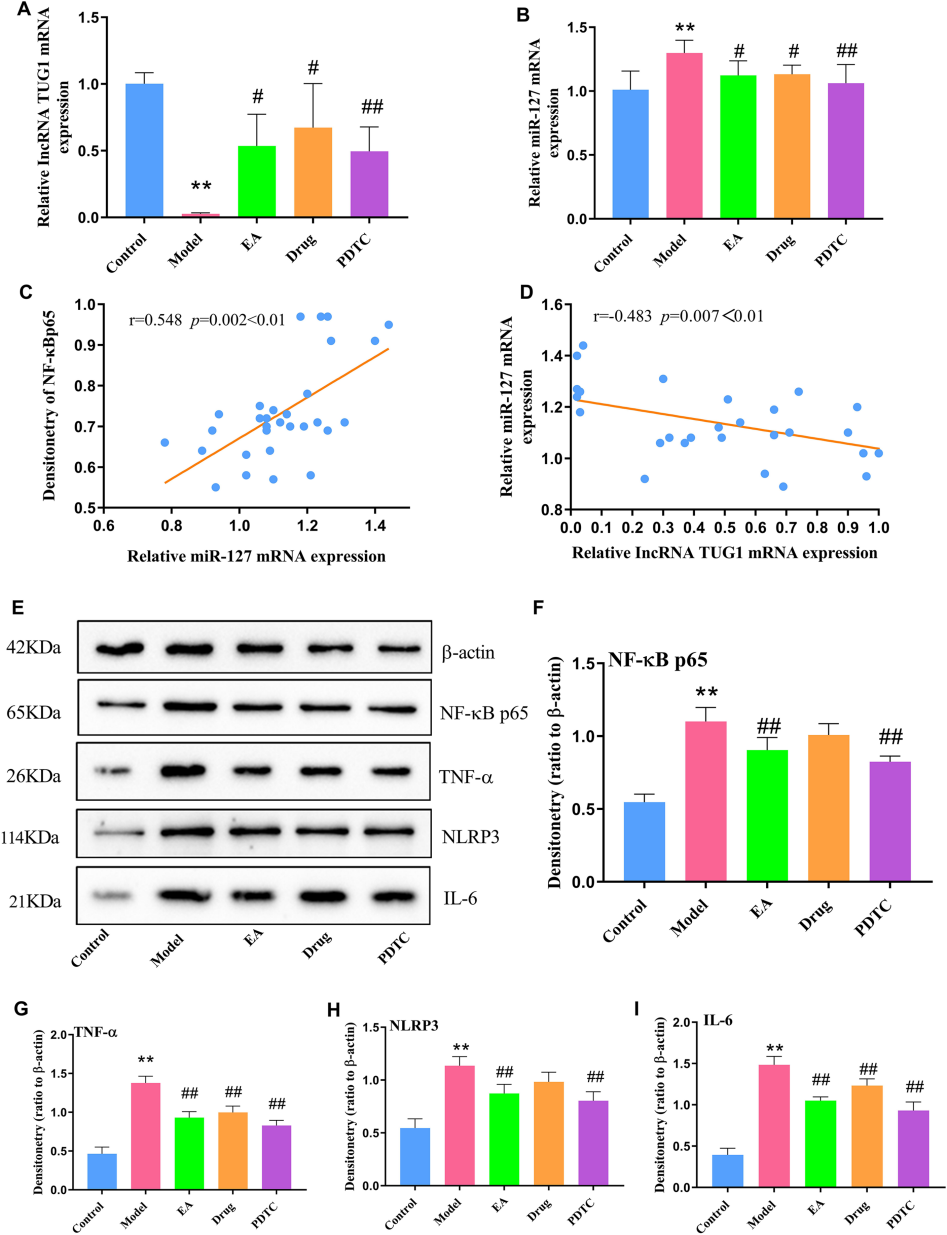
There is a correlation between lncRNA TUG1 and miR-127, miR-127 and NF-κ B in the colon of IBS-D rats, and they are regulated by electroacupuncture (n = 6). **(A-B)** Expression of lncRNA TUG1 **(A)** and miR-127 **(B)** in rat colon tissue detected using RT–qPCR. **(C-D)** Scatter plot of scatter plot of miR-127 and NF-κB in the colon **(C)** and lncRNA TUG1 and miR-127 **(D)**, showing a regression line with the *r* value representing the Pearson coefficient. **(E-I)** The protein expression of NF-κB P65 **(F)**, TNF-α **(G)**, NLRP3 **(H)**, and IL-6 **(I)** was detected using Western blotting**(E)**. ^**^p < 0.01 versus the control group, ^#^p < 0.05, ^##^p < 0.01 *vs* the model group.

BiBiServ RNAhybrid predicted that rno-lncRNA TUG1-WT had binding sites with rno-miR-127-3p, and rno-miR-127-3p had binding sites with rno-NF-KB p65-WT (**Figures 3A, B**). Next, we used 293T cells for the dual luciferase assay, and the results showed that rno-miR-127-3p significantly downregulated the luciferase expression of rno-lncRNA TUG1-WT (*P* < 0.01) (**Figure 3C**). In addition, rno-miR-127-3p significantly downregulated the expression of luciferase in rno-NF-KB P65-WT (*P* < 0.01) (**Figure 3D**). These findings indicate that rno-lncRNA TUG1-WT is capable of binding to rno-miR-127-3p, and rno-miR-127-3p can bind to rno-NF-KB p65-WT. Compared with the NC group, rno-miR-127-3p failed to downregulate luciferase expression of rno-lncRNA TUG1-MUT, and rno-NF-KB P65-MUR (*P* > 0.05) (**Figures 3C, D**), indicating that the mutation was successful. Luciferase reporter assays confirm direct binding capability between miR-127-3p and the TUG1 target sequence (**Figure 3C**). Combined with inverse correlation of endogenous expression levels in colon tissues (**Figures 4A, B**), these data support a competitive endogenous RNA mechanism whereby TUG1 sponges miR-127-3p.

#### 3.2.2 EA promotes colon LncRNA TUG1 and inhibits MiR-127-3p expression in IBS-D rats

The relative expression level of lncRNA TUG1 was evaluated by RT–qPCR and the results showed that the model group had significantly lower levels of lncRNA TUG1 expression in colon tissues than the control group *(P* < 0.01). After the treatment, the EA and drug groups exhibited significantly higher levels of lncRNA TUG1 expression in colon tissues than the model group (*P* < 0.05) (**Figure 4A**). The relative expression level of miR-127-3p in colon tissues was significantly higher in the model group than in the control group (*P* < 0.01). The relative expression level of miR-127-3p in the colon tissues of rats was lower in the EA and drug groups than in the model group (*P* < 0.05) (**Figure 4B**).

#### 3.2.3 MiR-127-3p and NF-κB p65 had a linear relationship

The levels of lncRNA TUG1 and miR-127-3p were initially determined using a scatter plot. Then, Pearson correlation analysis showed that the lncRNA TUG1 levels were negatively correlated with miR-127-3p levels (–1 < *R* < 0, *P* < 0.01). In addition, the scatter diagram revealed that miR-127-3p and NF-κB p65 had a linear relationship (**Figure 4C**). The bivariate Spearman correlation analysis showed a positive correlation between miR-127-3p levels and NF-κB p65 protein expression (0 < *r* < 1, *P* < 0.01) (**Figure 4D**).

### 3.3 EA inhibits NF-κB p65-driven inflammation by rescuing IκBα and A20 from miR-127-3p-mediated repression

#### 3.3.1 EA inhibits the expression of NF-κB P65, TNF-α, NLRP3, and IL-6 in serum of IBS-D rats

The protein expression levels were assessed by ELISA and the results showed that the protein expression levels of NF-κB P65, TNF-α, NLRP3, and IL-6 were significantly higher in the model group than in the control group (*P* < 0.01). However, after the treatment, the levels of NF-κB P65, TNF-α, NLRP3, and IL-6 were significantly lower in the EA, drug, and PDTC groups than in the model group (*P* < 0.01) (**Figures 3E–H**).

#### 3.3.2 EA inhibits the expression of NF-κB P65, TNF-α, NLRP3, and IL-6 in colon of IBS-D rats

Western blotting was used to examine the protein expression levels. Consistent with the ELISA findings, the protein expression levels of NF-κB P65, TNF-α, NLRP3, and IL-6 in the colon of the model group were significantly higher than those of the control group (*P* < 0.01). However, after treatment, the protein expression levels of NF-κB P65, TNF-α, NLRP3, and IL-6 were significantly lower in the EA, PDTC groups than in the model group (*P* < 0.01). The levels of TNF - α and IL-6 in the drug group were significantly reduced compared to the model group. (*P* < 0.01). (**Figures 4E–I**).

**Figure 5:**
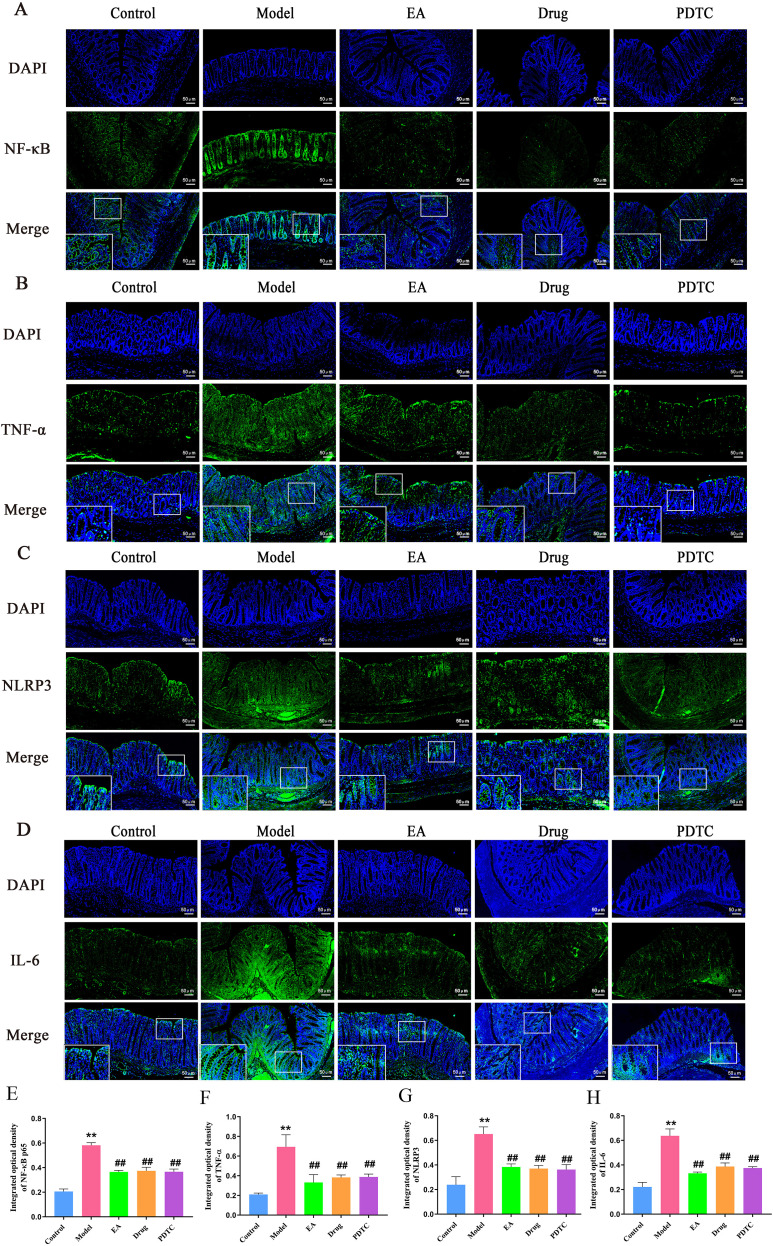
Electroacupuncture can reduce the expression of NF - κ B P65 and related inflammatory factors in the colon of IBS-D rats (n = 6). **(A–D)** Protein levels of NF-κB P65 **(A)**, TNF-α **(B)**, NLRP3 **(C)**, and IL-6 **(D)** in the colon tissues of rats in each group detected by immunofluorescence staining. **(E-H)** Average absorbance values of NF-κB P65 **(E)**, TNF-α **(F)**, NLRP3 **(G)**, and IL-6 **(H)** in the colon tissues of rats. ^**^p < 0.01 versus the control group; ^##^p < 0.01 versus the model group.

Immunofluorescence staining was used to detect the protein expression of NF-κB P65, TNF-α, NLRP3, and IL-6 in colon tissues. NF-κB P65 was mainly expressed in the nucleus (**Figure 5A**), TNF-α was mainly expressed in the cytomembrane (**Figure 5B**), NLRP3 was expressed in the cytoplasm (**Figure 5C**), and IL-6 was expressed in the outer membrane (**Figure 5D**). The average absorbance values of NF-κB P65, TNF-α, NLRP3, and IL-6 in the colon tissue were significantly higher in the model group than in the control group (*P* < 0.01). However, after treatment, the average absorbance values of NF-κB P65, TNF-α, NLRP3, and IL-6 in the EA, drug, and PDTC groups were significantly lower than those in the model group (*P* < 0.01) (**Figures 5E–H**).

**Figure 6:**
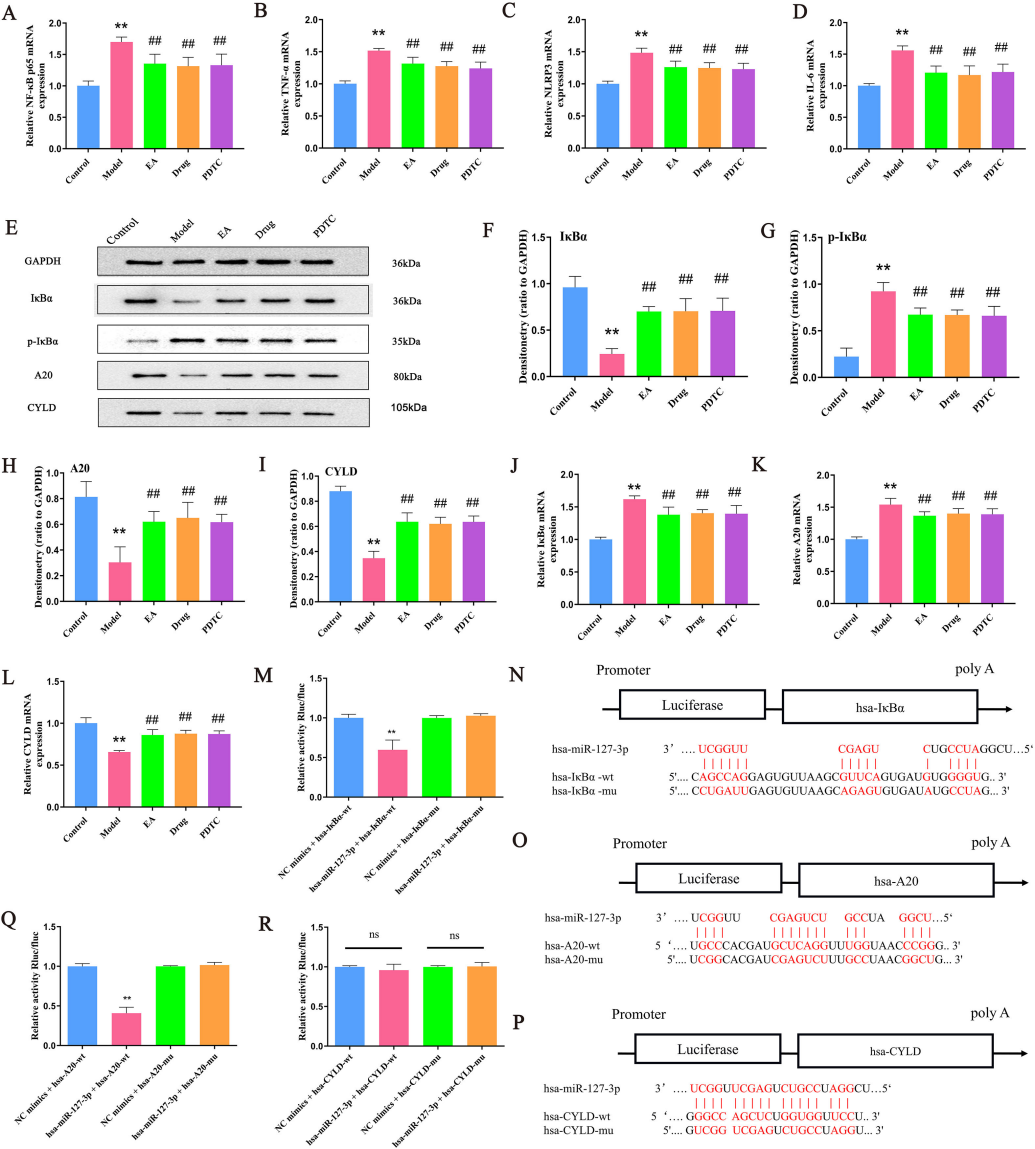
rno-miR-127-3p indirectly mediates significant activation of NF-κB in the colon tissue of IBS-D rats by suppressing its negative regulators (n = 6). **(A–D)** Expression of NF-κB P65 mRNA**(A)**, TNF-α mRNA **(B)**, NLRP3 mRNA **(C)**, and IL-6 mRNA **(D)** in rat colon tissue detected using RT–qPCR. **(E-I)** The protein expression of IκBα **(F)**, p-IκBα **(G)**, A20 **(H)**, and CYLD **(I)** in the colon tissues was detected using Western blotting **(E)**. **(J-L)** Expression of IκBα mRNA**(J)**, A20 mRNA **(K)**, and CYLD mRNA **(L)** in rat colon tissue detected using RT–qPCR. ^**^p < 0.01 versus the control group; ^##^p < 0.01 versus the model group. **(M–R)** Dual-luciferase reporter assays demonstrated that rno-miR-127-3p directly targets and binds to the 3’UTR regions of negative regulators IκBα **(M, N)**, and A20 **(O, Q)**, thereby suppressing their expression. There is no binding interaction with CYLD **(P, R)**. **p < 0.01 compared with the NC group.

The mRNA levels of *NF-κB P65, TNF-α, NLRP3*, and *IL-6* were quantified by RT-qPCR and were found to mirror the trends of their corresponding proteins, as observed by ELISA and Western blot (**Figures 6A–D**).

#### 3.3.3 rno-miR-127-3p sustains NF-κB p65 activation via post-transcriptional silencing of negative regulators IκBα and A20

Luciferase reporter assays confirmed a direct interaction between rno-miR-127-3p and the 3′-untranslated region (3′UTR) of NF-κB p65, and both p65 mRNA and protein were elevated in the model group. Because miRNAs classically repress their targets (36, 37), this apparent up-regulation was paradoxical. We therefore quantified the NF-κB p65 negative regulators Inhibitor of κB-α(IκBα), A20 (tumor necrosis factor-α induced protein 3, TNFAIP 3) and Cylindromatosis (CYLD). In the model group, protein levels of all three inhibitors were concordantly decreased (*P* < 0.01, **Figures 6F–I**). mRNA levels were divergent, IκBα and A20 were significantly increased, whereas CYLD mRNA was reduced (*P* < 0.01, **Figures 6J–L**). This mRNA–protein discordance was completely reversed by EA, Drug treatment, or PDTC. Subsequent luciferase assays demonstrated that rno-miR-127-3p directly bound to and suppressed the 3′UTRs of IκBα and A20, but had no appreciable effect on CYLD (**Figures 6M–R**). Collectively, rno-miR-127-3p relieves the brake on NF-κB p65 by silencing IκBα and A20, thereby indirectly sustaining NF-κB p65 activation.

### 3.4 EA restores colonic epithelial barrier integrity and tight junction architecture

#### 3.4.1 EA improves the ultrastructure of colonic mucosal epithelial cells in IBS-D rats

**Figure 7:**
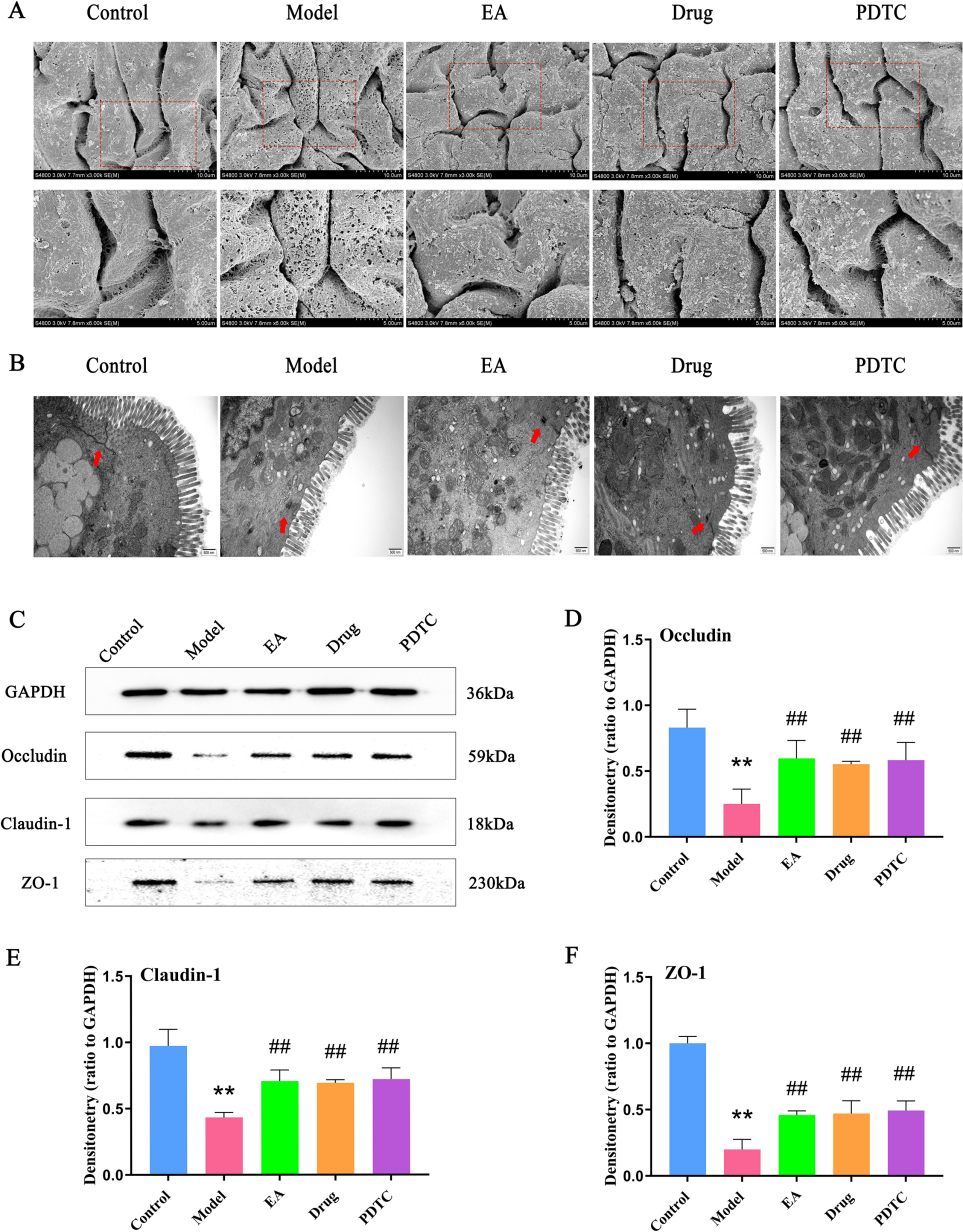
Electroacupuncture improves the ultrastructure of colonic mucosal epithelial cells in IBS-D rats (n = 6). **(A)** Comparison of the ultrastructure of colon mucosa in different groups of rats (scanning electron microscopy). The locally enlarged area refers to the location where the morphology of the colon mucosa changes significantly. **(B)** Comparison of the ultrastructure of colonic mucosal epithelial cells in different groups of rats (transmission electron microscopy). The red arrow points to the tight connections between cells. **(C–E)** The protein expression of Occludin **(D)**, Claudin-1 **(E)**, and ZO-1 **(F)** in the colon tissues was detected using Western blotting **(C)**. ^**^p < 0.01 versus the control group; ^##^p < 0.01 versus the model group.

Scanning electron microscopy was used to observe the ultrastructure of colon mucosa in each group of rats. The colonic mucosa in the control group exhibited regular morphology, characterized by orderly arranged villi and homogeneous density. In contrast, the colonic mucosa in the model group displayed irregular features, including shortened and sparsely distributed villi with heterogeneous density. Compared to the model group, the colon mucosa in the EA, drug, and PDTC groups exhibited greater regularity. The microvilli were increased in number, elongated, arranged in a more orderly fashion, and demonstrated a more uniform density (**Figure 7A**).

Transmission electron microscopy was used to observe the ultrastructure of colonic mucosal epithelial cells in each group of rats. In the control rats, colonic mucosal epithelial cells displayed regular morphology with well-organized microvilli and intact structures, normal intercellular spaces, and undamaged tight junctions. By contrast, model group rats exhibited disrupted epithelial cell morphology, characterized by sparse, irregular, shortened, and thickened microvilli, along with compromised tight junctions and widened intercellular gaps. The cell morphology of the EA, drug, and PDTC groups was slightly disrupted, with increased and elongated microvilli compared to the model group, arranged neatly, and damaged tight junctions (**Figure 7B**).

#### 3.4.2 EA promotes the expression of occludin, claudin-1, and ZO-1 in colon of IBS-D rats

**Figure 8:**
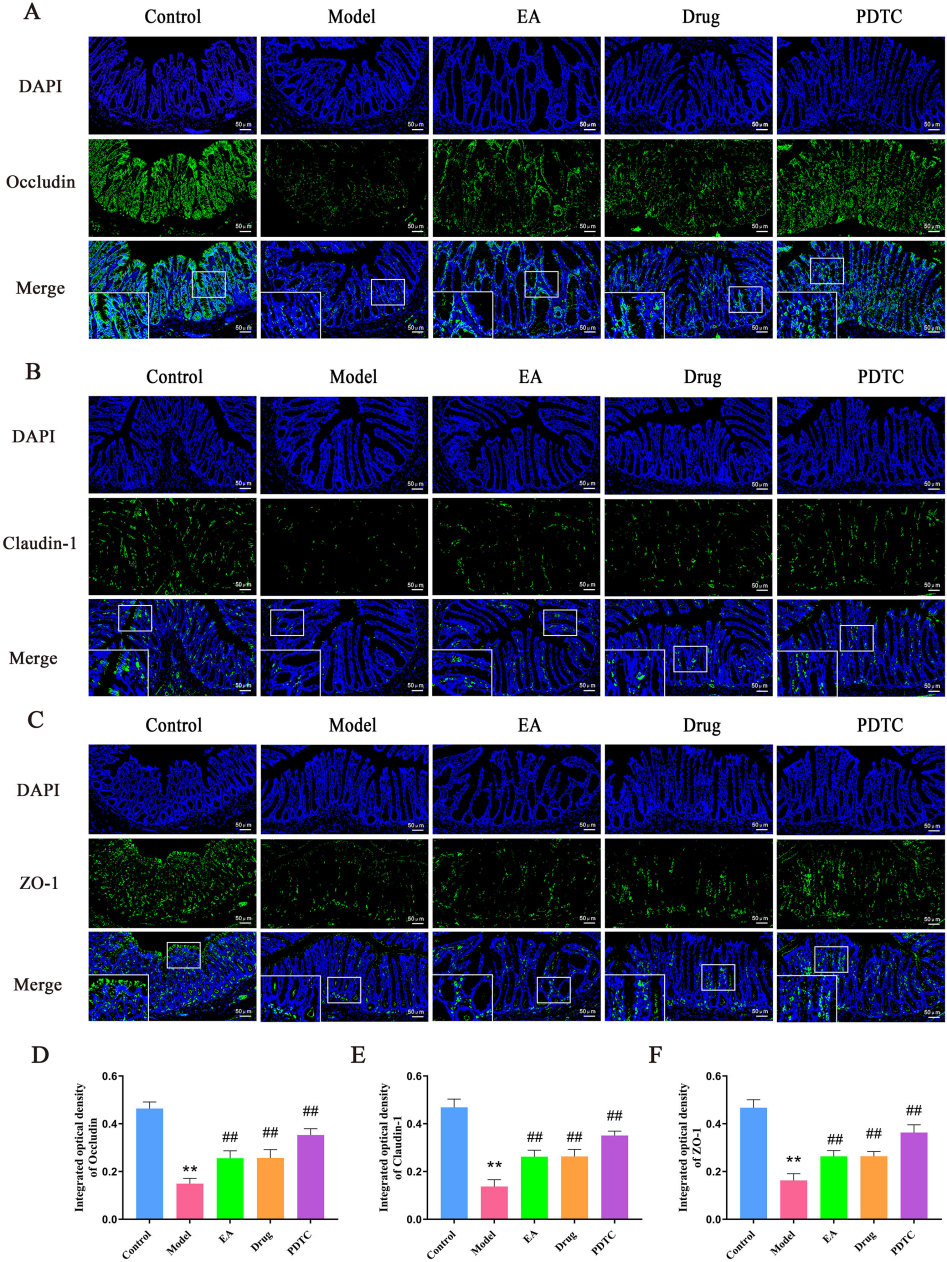
Electroacupuncture upregulates the expression of tight junction proteins in the colonic mucosa of IBS-D rats (n = 6). **(A–C)** Protein levels of Occludin **(A)**, Claudin-1 **(B)**, and ZO-1 **(C)** in the colon tissues of rats in each group were detected by immunofluorescence staining. **(D–F)** Average absorbance values of Occludin **(D)**, Claudin-1 **(E)**, and ZO-1 **(F)** in the colon tissues of rats. ^**^p < 0.01 versus the control group; ^##^p < 0.01 versus the model group.

Western blotting (**Figure 7C**) and Immunofluorescence Staining (**Figures 8A–C**) were used to detect the expression of tight junction proteins occludin, claudin-1, and ZO-1 in colon tissues. occludin and claudin-1 were mainly expressed in the cytomembrane (**Figures 8A, B**), and ZO-1 was expressed in the cytoplasm (**Figure 8C**). The expression of occludin, claudin-1, and ZO-1 in the colon tissue was significantly lower in the model group than in the control group (*P* < 0.01). However, after treatment, the expression of occludin, claudin-1, and ZO-1 in the EA, drug, and PDTC groups was significantly higher than that in the model group (*P* < 0.01) (**Figures 7D–F**, **8D–F**).

## 4 Discussion

The abnormal expression of inflammatory mediators has been demonstrated to significantly influence the occurrence, progression, and outcome of IBS (38). Therefore, low-level intestinal mucosal inflammation is regarded as a crucial pathophysiological mechanism in IBS and a pivotal factor in its treatment (38–40). The present study established an IBS-D rat model by combining maternal-infant separation, acetic acid enema, and wrap restraint stress. Successful model preparation was confirmed by decreased minimum water injection volume at AWR score 3 and increased loose stool rate, corresponding to the cardinal symptoms of IBS-D, namely diarrhea and visceral hypersensitivity. Following EA treatment, the minimum water injection volume at AWR score 3 increased and the loose stool rate decreased, suggesting that EA intervention ameliorates diarrhea and visceral hypersensitivity in IBS-D rats. Mechanistically, EA upregulated colonic lncRNA TUG1 expression, downregulated the miR-127-3p/NF-κB p65 axis, and reduced the expression of inflammation-related proteins TNF-α, NLRP3, and IL-6 in both serum and colon. Concurrently, colonic tight junction proteins occludin, claudin-1, and ZO-1 were upregulated, and colonic mucosal epithelial barrier impairment was repaired. These findings imply that EA ultimately exerts biological effects in alleviating low-grade inflammation in the IBS-D colon. IBS-D is characterized by the absence of structural or biochemical abnormalities, with chronically persistent symptoms (41). Visceral hypersensitivity, abdominal pain, and diarrhea constitute the main clinical manifestations, driven by low-grade intestinal inflammation, immune activation, gut microbiota alterations, and intestinal barrier damage (42). Despite the expanding therapeutic repertoire for IBS-D (42–44), long-term outcomes remain unsatisfactory. EA has gained widespread recognition due to its remarkable efficacy and favorable safety profile (8, 19, 45, 46); however, its underlying molecular mechanisms remain to be fully elucidated.

To clarify the mechanism by which EA restores intestinal integrity, our data support a model in which EA exerts effects through immune-mediated pathways: upregulated lncRNA TUG1 expression in the colonic mucosa sponges miR-127-3p, thereby relieving post-transcriptional suppression of NF-κB p65 negative regulators (IκBα and A20). This inhibition of NF-κB p65 signaling reduces pro-inflammatory cytokine release (TNF-α, NLRP3, IL-6), subsequently creating a microenvironment permissive for tight junction protein restoration and epithelial barrier repair. This indirect mechanism is supported by three lines of evidence: (i) the temporal sequence of molecular changes preceding morphological barrier recovery; (ii) the parallel restoration of tight junction proteins by the NF-κB p65 inhibitor PDTC, which acts solely through immune modulation; and (iii) the direct binding interactions confirmed by dual-luciferase assays linking TUG1→miR-127-3p→IκBα/A20→NF-κB p65. NF-κB p65 plays a crucial role in regulating intestinal mucosal immune responses (47). Upon encountering diverse stimuli, the intestinal mucosa transmits signals through pattern recognition receptors such as TLRs, activating the NF-κB p65 pathway (47, 48). Phosphorylation and nuclear translocation of NF-κB p65 induce downstream gene expression, directly activating NLRP3, TNF-α, and IL-6, which exacerbate intestinal mucosal barrier damage and visceral hypersensitivity (49, 50). miRNAs are crucial post-transcriptional modulators influencing cellular differentiation, growth, and programmed cell death (51, 52). Accumulating evidence reveals that miRNAs play important roles in gastrointestinal diseases, affecting IBS-D pathogenesis through intestinal mucosal barrier function, visceral hypersensitivity, low-grade inflammation, and gut microbiota (53, 54). Specifically, miR-127-3p promotes macrophage inflammatory responses (55), activates the NF-κB p65 signaling pathway, and induces upregulation of pro-inflammatory cytokines including TNF-α, IL-6, and IL-1β (23, 24, 56). However, canonical miRNA biology holds that miRNAs repress gene expression by binding the 3′UTR of target transcripts, blocking translation or promoting degradation—a mechanism seemingly contradictory to the observed upregulation of both NF-κB p65 mRNA and protein in the model group (36, 37). Our data resolve this paradox by demonstrating that rno-miR-127-3p sustains NF-κB p65 activation indirectly through post-transcriptional silencing of its negative feedback regulators IκBα and A20. Although IκBα and A20 mRNA were transcriptionally upregulated—consistent with NF-κB p65-driven compensatory feedback—their corresponding proteins were markedly reduced. Dual-luciferase reporter assays confirmed direct binding of rno-miR-127-3p to the 3′UTRs of IκBα and A20, resulting in translational repression; no functional binding site was detected in CYLD, whose mRNA decline likely reflects alternative degradation pathways. Collectively, rno-miR-127-3p relieves NF-κB p65 self-braking by suppressing IκBα and A20 translation, thereby perpetuating mucosal inflammation in IBS-D. TUG1, a 7.1 kb non-coding RNA, is intimately associated with inflammatory responses (22, 57, 58). The present study demonstrated that lncRNA TUG1 associates with miR-127-3p through dual-luciferase reporter assays. In IBS-D rats, decreased TUG1 levels upregulated miR-127-3p, activated the NF-κB p65 pathway, and triggered inflammation. Following EA, increased TUG1 levels altered miR-127-3p and NF-κB p65 expression, inhibiting inflammation. Correlation analysis demonstrated inverse associations between lncRNA TUG1 and both miR-127-3p and NF-κB p65 expression. These findings reveal that EA alleviates inflammatory responses in IBS-D by modulating TUG1 expression, negatively regulating miR-127-3p, and influencing NF-κB p65, TNF-α, NLRP3, and IL-6 expression.

The restoration of intestinal barrier integrity observed in this study—evidenced by increased tight junction protein expression (occludin, claudin-1, ZO-1) and improved mucosal ultrastructure—is best interpreted as a consequence of reduced low-grade inflammation rather than direct transcriptional activation of epithelial junction components by EA. This interpretation aligns with the established understanding that chronic inflammation disrupts tight junction architecture in IBS-D (59), and that resolution of inflammatory signaling permits spontaneous barrier recovery. Notably, both EA and the NF-κB p65 antagonist PDTC comparably restored tight junction protein levels despite differing primary mechanisms (neuromodulation versus direct NF-κB p65 inhibition), reinforcing that barrier restoration occurs downstream of inflammatory suppression. We explicitly acknowledge that direct neural regulation of epithelial permeability—while plausible given EA’s known neuromodulatory effects—was not mechanistically investigated in this study and represents an important avenue for future research. In this experiment, IBS-D rats exhibited elevated levels of TNF-α, NLRP3, and IL-6 alongside colonic mucosal barrier impairment, indicative of augmented intestinal inflammatory responses. Following EA treatment, serum and colonic levels of these inflammatory mediators were significantly reduced, the damaged colonic mucosa was repaired, and intestinal inflammation was attenuated. These findings, combined with previous research (60–62), suggest that EA inhibits inflammatory factor expression, reduces inflammatory effects, repairs colonic mucosal epithelial barrier impairment, and directly or indirectly reduces diarrhea and visceral hypersensitivity in IBS-D rats. NLRP3 activates proinflammatory caspase-1, facilitating interleukin maturation and driving inflammation progression. EA inhibited NLRP3 overactivation, maintained intestinal homeostasis, and was beneficial in suppressing intestinal mucosal inflammation (63, 64), consistent with earlier investigations (65).

Interestingly, both EA and rifaximin interventions converged on upregulating colonic TUG1 levels (**Figure 4A**). We postulate that this common effect stems not from a shared initial trigger, but from a shared downstream consequence: amelioration of the local inflammatory milieu. The chronic NF-κB p65-driven inflammatory state in IBS-D may actively suppress TUG1 expression. Both EA and rifaximin, by mitigating this inflammation through potentially distinct primary mechanisms—neuromodulation versus microbiota alteration, respectively—may relieve transcriptional repression on TUG1, allowing restoration of its expression. This represents a convergent point in their mechanisms of action, warranting future investigation into the specific transcription factors governing TUG1’s response to inflammatory signals. While our study supports comparable efficacy among EA, rifaximin, and PDTC as monotherapies, the potential for synergistic combination remains unexplored. The convergent TUG1 upregulation via distinct primary mechanisms suggests that combination therapy might achieve superior outcomes through pathway complementarity. Future factorial designs comparing monotherapies versus combined EA plus sub-therapeutic drug doses would directly test this hypothesis. While gut microbiota dysbiosis is established in IBS-D pathophysiology, our findings suggest that EA modulation of the TUG1/miR-127-3p/NF-κB p65 axis likely occurs through microbiome-independent neuromodulatory mechanisms. The comparable efficacy of EA and rifaximin—despite their distinct primary targets—indicates that TUG1 upregulation can be achieved through multiple upstream pathways converging on NF-κB p65 suppression. Nevertheless, we cannot exclude that EA-induced neural activation may secondarily influence microbial composition or metabolite production, nor whether an intact microbiota is required for full EA efficacy. The ST25/ST37 acupoint selection was based on traditional “He-Mu” principles for harmonizing intestinal function, and contemporary studies demonstrate that EA at these points modulates visceral hypersensitivity through spinal and supraspinal mechanisms (17, 18). We acknowledge that this study investigated EA as an integrated modality; thus, the independent contributions of acupoint specificity, needle insertion, and electrical stimulation were not isolated. Future studies employing sham-acupuncture controls, non-acupoint stimulation, and varying electrical parameters are needed to parse these components. However, the comparable suppression of NF-κB p65-driven inflammation by EA and PDTC indicates that the identified molecular anti-inflammatory mechanism is robust, supporting the clinical relevance of the EA protocol as tested.

Our study establishes the TUG1/miR-127-3p/NF-κB p65 axis as a critical pathway for EA’s anti-inflammatory effects in IBS-D; however, we recognize that this represents one component of a complex therapeutic mechanism. Specifically, we did not directly test whether EA acts on epithelial cells through neural-mediated pathways independent of immune modulation. Whether these neural pathways converge on the TUG1/miR-127-3p/NF-κB p65 axis—or operate in parallel—the mechanism linking peripheral EA stimulation to colonic TUG1 upregulation remains undefined. We propose two hypotheses. First, EA may derepress TUG1 transcription indirectly via neuro-immune modulation: by suppressing NF-κB p65-driven inflammation through spinal–supraspinal pathways, EA relieves chronic inflammatory repression of TUG1. Second, TUG1 restoration may represent a convergent downstream consequence of NF-κB p65 inhibition rather than a direct EA target; the parallel upregulation of TUG1 by EA and rifaximin—despite their distinct primary mechanisms—supports this interpretation. These hypotheses remain speculative; future studies using denervation models, enteric neuron–epithelium co-cultures, or promoter-binding assays will be required to elucidate the precise signaling linkage. We further acknowledge that the present study measured bulk tissue levels of NF-κB p65, NLRP3, TNF-α, and IL-6 without delineating their specific cellular origins. While these mediators are broadly expressed across epithelial, immune, and stromal populations, our data cannot distinguish whether the observed changes reflect altered mast cell activity, macrophage infiltration, or epithelial NF-κB signaling. Future studies employing cell-type-specific approaches will be required to dissect these respective contributions. We also acknowledge that sex was not analyzed as a biological variable in this study; future investigations with adequately powered cohorts are warranted to determine whether the TUG1/miR-127-3p/NF-κB p65 axis and EA responsiveness differ between sexes. Finally, although our data demonstrate coordinate regulation of this axis by EA, functional rescue experiments are required to establish that TUG1 upregulation is necessary and sufficient for EA-mediated therapeutic effects. These include siRNA-mediated TUG1 knockdown combined with EA treatment and lentiviral miR-127-3p overexpression in EA-treated animals to confirm the causal role of this axis in therapeutic efficacy; future studies using germ-free models or antibiotic depletion will clarify the microbiome-dependency of the observed molecular effects.

In conclusion, this study demonstrates that EA decreases low-grade inflammation in the IBS-D intestine through lncRNA TUG1-mediated downregulation of miR-127-3p, thereby inhibiting NF-κB p65 and reducing inflammatory factor release to repair colonic mucosal epithelial barrier impairment. In contrast to previous studies on EA treatment of IBS, the present investigation employed dual-luciferase detection to validate the regulatory relationship between lncRNA TUG1 and miR-127-3p, demonstrating for the first time that EA reduces low-grade intestinal inflammation in IBS-D at least in part through modulation of the miR-127-3p/NF-κB p65 axis. These findings provide novel molecular evidence supporting acupuncture treatment of IBS-D. A key mechanism of EA in IBS treatment is its ability to suppress mild intestinal inflammation indirectly, thereby restoring intestinal barrier integrity as a downstream consequence of reduced NF-κB p65-driven cytokine release. Nevertheless, the exact mechanisms through which EA exerts its influence on IBS-D-related inflammation remain highly intricate and necessitate more in-depth exploration.

## 5 Conclusion

**Figure 9:**
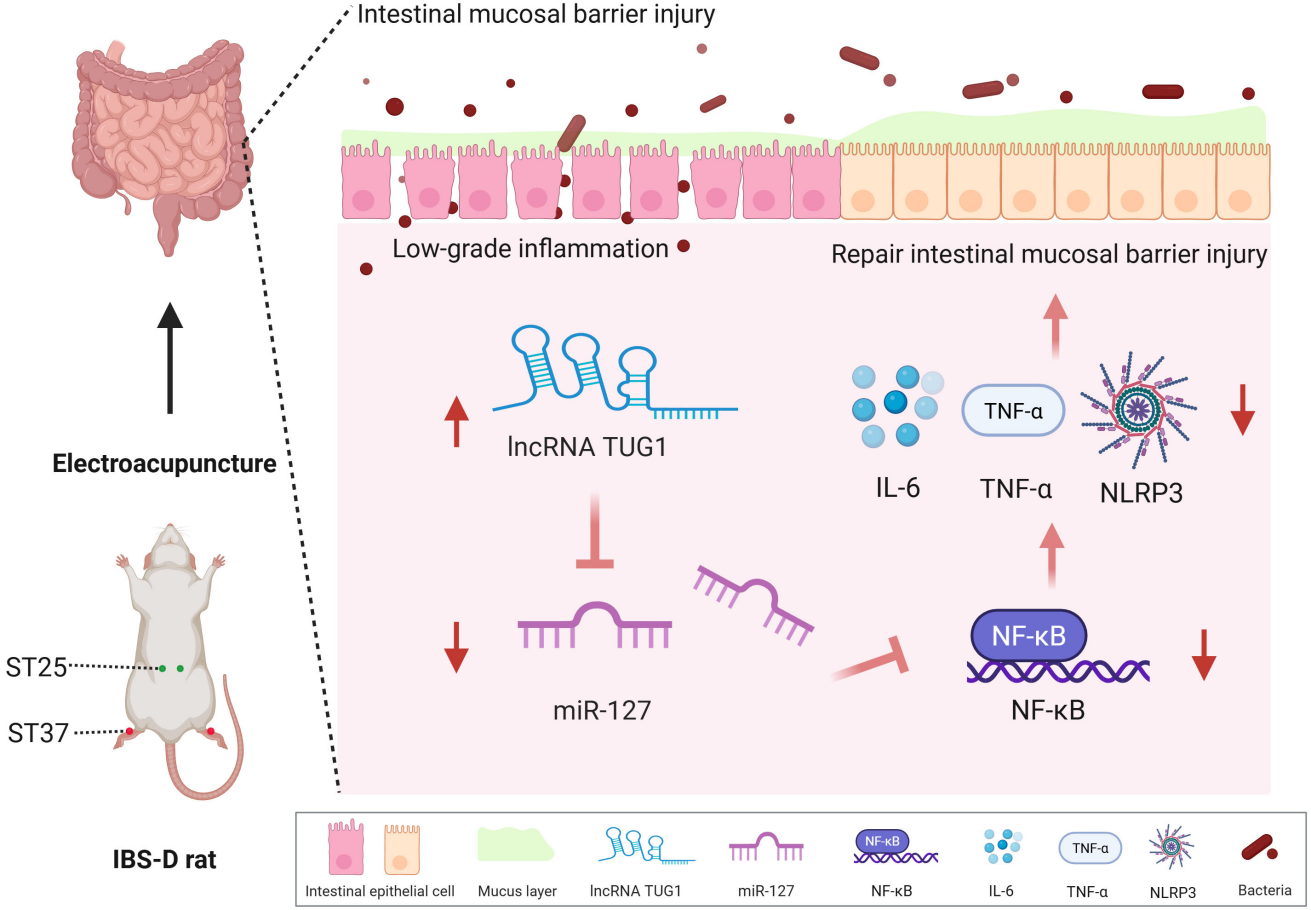
Mechanisms of EA-mediated low-grade colonic inflammation via the upregulation of lncRNA TUG1 to suppress the miR-127/NF-κB axis in IBS-D rats. Created in BioRender. LI, K. (2025) https://BioRender.com/olqzrfx.

EA attenuates low-grade colonic inflammation and visceral hypersensitivity in IBS-D, at least in part through upregulation of lncRNA TUG1, which is associated with suppression of the miR-127-3p/NF-κB p65 axis and post-transcriptional derepression of IκBα and A20. This mechanism is consistent with reduced downstream inflammatory mediators (TNF-α, NLRP3, IL-6) and restoration of intestinal barrier integrity. These findings support a contributory role for the lncRNA TUG1/miR-127-3p/NF-κB p65 axis in IBS-D pathogenesis, provide novel molecular evidence consistent with EA treatment effects, and suggest this axis as a candidate therapeutic target. Functional rescue experiments are warranted to establish the necessity and sufficiency of this axis in mediating EA efficacy (**Figure 9**).

## Data Availability

The original contributions presented in the study are included in the article/**Supplementary Material**. Further inquiries can be directed to the corresponding author.
