# Supplementary material for: Electroacupuncture-mediated lncRNA TUG1 regulates the miR-127-3p/NF-κB p65 axis to inhibit IBS-D low-grade intestinal inflammation
Source: Front Immunol. 2026 Jul 28;17:1764684. doi: 10.3389/fimmu.2026.1764684 (PMC13457141; doi:10.3389/fimmu.2026.1764684)
Supplement: Supplementary file 1 [file Table1.docx]

**Table S1** The instrument and reagent specifications in vivo experiments.

| REAGENT OR RESOURCE | SOURCE | IDENTIFIER |
| --- | --- | --- |
| Antibodies | | |
| Anti mouse TNF-α antibody | Santa Cruz Biotechnology | Cat# sc-52746, RRID: AB_630341 |
| Anti mouse IL-6 antibody | Santa Cruz Biotechnology | Cat# sc-28343, RRID: AB_627805 |
| Anti Rabbit NF-κB P65 antibody | Abcam | Cat# ab16502, RRID: AB_443394 |
| Anti Rabbit NLRP3 antibody | Bioss | Cat# bs-6655R, RID: AB_11102267 |
| Anti Rabbit ZO-1 | Proteintech | Cat# 21773-1-AP, RRID: AB_10733242 |
| Anti Rabbit Occludin | ServiceBio | Cat# GB111401, RRID: AB_2938979 |
| Anti Rabbit Claudin-1 | Abcam | Cat# ab211737, RRID: AB_3082989 |
| Anti Rabbit IκBα | Abcam | Cat# ab32518, RRID: AB_733068 |
| Anti Rabbit p-IκBα | Abcam | Cat# ab133462, RRID: AB_2801653 |
| Anti mouse A20 | Proteintech | Cat# 66695-1-Ig, RRID: AB_2882048 |
| Anti Rabbit CYLD | bioss | Cat# bs-2756R, RRID: AB_10856273 |
| Anti mouse GAPDH | Zsbio | Cat# TA-08, RRID: AB_2747414 |
| Horseradish enzyme labeled goat anti-mouse IgG (H+L) | ZSGB-Bio | Cat# ZB-2305, RRID: AB_2747415 |
| Horseradish enzyme labeled goat anti-rabbit IgG (H+L) | ZSGB-Bio | Cat# ZB-2301, RRID: AB_2747412 |
| Anti mouse Occludin antibody | Santa Cruz Biotechnology | Cat# sc-133256, RRID: AB_2156317 |
| Anti Rabbit Claudin-1 antibody | Bioss | Cat# bs-1428R, RRID: AB_10856676 |
| Anti Rabbit ZO-1 antibody | Proteintech | Cat# 82870-7-RR, RRID: AB_3670590 |
| Cy3-labeled goat anti-mouse IgG (H+L) | Beyotime | Cat# A0521, RRID: AB_2923334 |
| Cy3 labeled goat anti-rabbit IgG (H+L) | Beyotime | Cat# A0516, RRID: AB_2893015 |
| Biological samples |  |  |
| samples from Rat | - | - |
| Chemicals, peptides, and recombinant proteins | | |
| Isoflurane | Lunan Better Pharmaceutical Co., Ltd. | Cat# H20020267 |
| Phosphate buffered solution (PBS) | Zs-BIO | Cat# ZLI-9062 |
| rifaximin | Alpha Sigma Co., Ltd. | Cat# H20181212 |
| PDTC | Shanghai Yuanye Biotechnology Co., Ltd. | Cat# S30342 |
| Pentobarbital | Shanghai Sph New Asia Pharmaceutical Co., Ltd. | Cat# SC-6017 |
| PmirGLO vectors | Promega | Cat# E1330 |
| Radio immunoprecipitation assay (RIPA) lysis buffer | Beyotime | Cat# P0013B |
| Trizol | Life technogies | Cat# 15596018 |
| 4',6-diamidino-2-phenylindole (DAPI) | Beyotime | Cat# P0126 |
| Glutaraldehyde solution | Ted Pella, Inc. | Cat# 2200218 |
| Critical commercial assays | | |
| Dual-luciferase detection kits | Promega | Cat# E1910 |
| Ready-to-Use Rapid Immunohistochemistry MaxVision™ HRP Kit (Mouse/Rabbit) | Fuzhou Maixin Biotech | Cat# KIT5030 |
| Experimental models: Cell lines | | |
| HEK-293T Cell | ICell Bioscience Inc | Cat# iCell-h237 |
| Experimental models: Organisms/strains | | |
| Sprague-Dawley (SD) rat, female | Animal Research Facility, Anhui Medical University | N/A |
| Oligonucleotides | | |
| Sense primer for lncRNA TUG1:  5'-TTATTCCCTCCTGCCATAC-3' | Sangon Biotech | N/A |
| Antisense primer for lncRNA TUG1:  5'-TTGGGTGATACTGTTGATTTAG-3' | Sangon Biotech | N/A |
| Sense primer for miR-127:  5'-GCTCGGATCCGTCTGAGC-3' | Sangon Biotech | N/A |
| Antisense primer for miR-127:  5'-AGTGCAGGGTCCGAGGTATT-3' | Sangon Biotech | N/A |
| RT primer for miR-127: 5'-GTCGTATCCAGTGCAGGGTCCGAGGTATTCGCACTGGATACGACAGCCAA-3' | Sangon Biotech | N/A |
| Sense primer for NF-κB p65:  5'- TCTACAGGCAGAAGGCGGAGGA -3' | Sangon Biotech | N/A |
| Antisense primer for NF-κB p65:  5'- TGGCGTCTGACACCACAGGTTC -3' | Sangon Biotech | N/A |
| Sense primer for TNF-α:  5'- GGGCCACCACGCTCTTCTGT -3' | Sangon Biotech | N/A |
| Antisense primer for TNF-α:  5'- GGCTACGGGCTTGTCACTCG -3' | Sangon Biotech | N/A |
| Sense primer for IL-6:  5'- GCCTTCTTGGGACTGATGTT -3' | Sangon Biotech | N/A |
| Antisense primer for IL-6:  5'- ACTGGTCTGTTGTGGGTGGT -3' | Sangon Biotech | N/A |
| Sense primer for NLRP3:  5'- ATGCCGTATCTGGTTGTGTT -3' | Sangon Biotech | N/A |
| Antisense primer for NLRP3:  5'- GACTTAGGAAGAGCTGAGCA -3' | Sangon Biotech | N/A |
| Sense primer for IκBα:  5'- GAAAATCTGGGGAAACTCAGC -3' | Sangon Biotech | N/A |
| Antisense primer for IκBα:  5'- TGTTAAATAGCCACACCCCG -3' | Sangon Biotech | N/A |
| Sense primer for A20:  5'- ACGACCTTTGCTTACTATGT -3' | Sangon Biotech | N/A |
| Antisense primer for A20:  5'- AATGGCAGAAACCTCCTC -3' | Sangon Biotech | N/A |
| Sense primer for CYLD:  5'- TTTTGCCAGCTAATGTTC -3' | Sangon Biotech | N/A |
| Antisense primer for CYLD:  5'- TACTGTCCTATGCTCCCT -3' | Sangon Biotech | N/A |
| Sense primer for β-actin:  5'- CCCATCTATGAGGGTTACGC -3' | Sangon Biotech | N/A |
| Antisense primer for β-actin:  5'-TTTAATGTCACGCACGATTTC -3' | Sangon Biotech | N/A |
| Software and algorithms | | |
| SPSS 26.0 | IBM | N/A |
| GraphPad Prism 9.5 | Graphpad Software Inc | https://www.graphpad.com/scientificsoftware/prism/ |
| ImageJ | National Institutes of Health | https://imagej.nih.gov/ij/ |
| CaseViewer 2.4 | 3DHISTECH Ltd. | https://www.3dhistech.com/ |
| Jetta JD801 morphological image analysis system | JEDA | http://www.jsjeda.com/ |
| BioRender | BioRender | https://www.biorender.com/ |
| Microsoft Office Microsoft 2019 | N/A | N/A |
| Zotero 7.0.16 | N/A | https://www.zotero.org/ |
| Photoshop Adobe 2023 | N/A | N/A |
| Other | | |
| Stainless steel acupuncture needle | Suzhou Tianxie Instrument Co., Ltd. | N/A |
| Electro-Acupuncture Device | Suzhou Medical Appliance Factory Ltd. | Cat# SDZ-Ⅱ |
